# PET/CT Based EGFR Mutation Status Classification of NSCLC Using Deep Learning Features and Radiomics Features

**DOI:** 10.3389/fphar.2022.898529

**Published:** 2022-04-27

**Authors:** Weicheng Huang, Jingyi Wang, Haolin Wang, Yuxiang Zhang, Fengjun Zhao, Kang Li, Linzhi Su, Fei Kang, Xin Cao

**Affiliations:** ^1^ School of Information Science and Technology, Northwest University, Xi'an, China; ^2^ National and Local Joint Engineering Research Center for Cultural Heritage Digitization, Xi'an, China; ^3^ Department of Nuclear Medicine, Xijing Hospital, Fourth Military Medical University, Xi'an, China

**Keywords:** radiomics, deep learning, EGFR, PET/CT, lung cancer

## Abstract

**Purpose:** This study aimed to compare the performance of radiomics and deep learning in predicting EGFR mutation status in patients with lung cancer based on PET/CT images, and tried to explore a model with excellent prediction performance to accurately predict EGFR mutation status in patients with non-small cell lung cancer (NSCLC).

**Method:** PET/CT images of 194 NSCLC patients from Xijing Hospital were collected and divided into a training set and a validation set according to the ratio of 7:3. Statistics were made on patients’ clinical characteristics, and a large number of features were extracted based on their PET/CT images (4306 radiomics features and 2048 deep learning features per person) with the pyradiomics toolkit and 3D convolutional neural network. Then a radiomics model (RM), a deep learning model (DLM), and a hybrid model (HM) were established. The performance of the three models was compared by receiver operating characteristic (ROC) curves, sensitivity, specificity, accuracy, calibration curves, and decision curves. In addition, a nomogram based on a deep learning score (DS) and the most significant clinical characteristic was plotted.

**Result:** In the training set composed of 138 patients (64 with EGFR mutation and 74 without EGFR mutation), the area under the ROC curve (AUC) of HM (0.91, 95% CI: 0.86–0.96) was higher than that of RM (0.82, 95% CI: 0.75–0.89) and DLM (0.90, 95% CI: 0.85–0.95). In the validation set composed of 57 patients (32 with EGFR mutation and 25 without EGFR mutation), the AUC of HM (0.85, 95% CI: 0.77–0.93) was also higher than that of RM (0.68, 95% CI: 0.52–0.84) and DLM (0.79, 95% CI: 0.67–0.91). In all, HM achieved better diagnostic performance in predicting EGFR mutation status in NSCLC patients than two other models.

**Conclusion:** Our study showed that the deep learning model based on PET/CT images had better performance than radiomics model in diagnosing EGFR mutation status of NSCLC patients based on PET/CT images. Combined with the most statistically significant clinical characteristic (smoking) and deep learning features, our hybrid model had better performance in predicting EGFR mutation types of patients than two other models, which could enable NSCLC patients to choose more personalized treatment schemes.

## Introduction

Lung cancer is the most dangerous cancer around the world, and its mortality is higher than all other cancers (Gansler et al., 2010; Bray et al., 2018). Among all lung cancers, non-small cell lung cancer (NSCLC) proves to be the most common histological subtype, which accounts for more than 80% (Ahmad and Gadgeel, 2016; Ettinger et al., 2018). For the patients with epidermal growth factor receptor (EGFR) gene mutations, epi-dermal growth factor receptor tyrosine kinase inhibitors (TKIs) have been increasingly used in the treatment and it is proved that it can significantly improve the survival time of patients (Zhu et al., 2019). Thus, it is significant to judge whether a patient has EGFR mutations before targeted therapy. At present, the commonly used gene detection methods are invasive, such as using tissue or cytological specimens. Because puncture biopsy can lead to serious complications, such as pneumothorax and hemoptysis, some patients can not complete the above invasive operations (Zhou et al., 2018; Liu et al., 2020). Therefore, it is urgent to develop a non-invasive, accurate, and simple gene mutation detection method.

Radiomics is a new field based on analyzing quantitative medical images, which aims at connecting the large-scale extracted medical imaging information with biological and clinical endpoints. With the continuous development of image analysis algorithms, data science research has the opportunity to turn to more personalized cancer treatment. Increasing evidence has proved that as a non-invasive method, radiomics can reveal the key components of tumor phenotype of lesions (Kirienko et al., 2018; Hassani et al., 2019; Kang et al., 2019; Sollini et al., 2019; Tu et al., 2019; Jiang et al., 2020). On the other hand, deep learning performance has been widely proved in computer vision in recent years. Convolutional neural network (CNN) is a typical artificial neural network in deep learning. It has achieved the most advanced image and video recognition and segmentation performance (Zhang et al., 2017; Singh et al., 2020; Cui et al., 2022). Radiomics and deep learning systems have been proved to have excellent performance in classifying, detecting, and segmenting lesions, and even predicting the risk of cancer on medical images (Hosny et al., 2019; Zheng et al., 2020; Castillo et al., 2021; Liu et al., 2021).

Previous studies focused on CT or MRI in the use of radiomics and deep learning to distinguish gene mutations (Li et al., 2018; Digumarthy et al., 2019; Lu et al., 2020; Wu et al., 2020; Zhang B. et al., 2020). Previous studies also proved that dual-mode PET/CT images can provide more information and improve the prediction accuracy than single-mode CT or MRI images. To compare the performance of radiomics and deep learning in predicting EGFR mutation status in patients with NSCLC based on PET/CT images, three models were established based on PET/CT images in this study, namely, radiomics model (RM), deep learning model (DLM), and hybrid model (HM), and they were compared the performance in identifying EGFR mutation status.

## Materials and Methods

### Patients Selection

This is a retrospective study of patients who underwent ^18^F-FDG PET/CT in Xijing Hospital from 2016 to 2021. Firstly, after excluding the patients 1) without obvious lung lesion, 2) without a documented pathological diagnosis, 3) with chemotherapy and/or radiotherapy before PET scans, 4) with poor PET/CT image quality, 194 patients 1) with a pathological or cytological diagnosis with NSCLC, 2) with complete ^18^F-FDG PET/CT imaging, 3) with a lesion diameter of >1 cm, 4) with EGFR detection and samples taken from primary NSCLC tissues, 5) without a history of other malignant tumors were included for further grouping. This study was approved by the medical ethics committee of Xijing Hospital (approval No: ky20173008-1).

### ^18^F-FDG PET/CT Acquisition and ROI Segmentation

All patients received ^18^F-FDG PET/CT scans (Biograph 40, Siemens, Germany) by following standard clinical protocols. Patients were required to fast for more than 6 h and blood glucose control within 7 mmol/L 5.55 MBq/kg of ^18^F-FDG was injected and scanned after 60 min. CT parameters were 120 keV, 110 mAs, 3–5 mm slice thickness. PET parameters were acquired for 5–7 beds, each bed was scanned for 2 min. The images were reconstructed using the ordered-subsets expectation-maximization algorithm with 4 iterations, 8 subsets, and matrix of 512 × 512.

Three-dimensional regions of interest (3D-ROIs) on the CT and PET images were respectively delineated by using MITK software (www.mitk.org). All the delineation work was done under the guidance of experienced PET/CT clinicians blinded to the pathological grouping. CT 3D-ROIs were manually delineated slice-by-slice around the lesions in CT images of lung window (WW: 1600HU, WL: 600HU). For PET segmentation, 3D-ROIs were semi-automatically drawn by referencing to the ROIs with a standard uptake value threshold of 40% using the “region grow” tool. For the lesions with anatomy boundary close to mediastinum or bronchus, CT images were used as a reference to determine the actual boundary in PET delineation.

### Radiomics Feature Extraction

Radiomics features for both CT images and PET images based on the 3D ROIs were extracted by using Pyradiomics (http://www.radiomics.io/pyradiomics.html) 3.0 version. Before the feature extraction, images were preprocessed using the default setting. To simplify the calculation of texture features, 25 binwidth was used for CT images and 0.1 binwidth for PET images to discretize the gray-level intensity. These features comprise first-order, shape, and texture features. The first-order feature describes the intensity distribution of CT and PET values in the volume of interest, such as median, energy, and skewness. The texture features include five classes: 1) gray-level co-occurrence matrix; 2) gray-level difference matrix; 3) gray-level run-length matrix; 4) gray-level size-zone matrix; 5) neighborhood gray-tone difference matrix. In addition, eight image filters (wavelet, lbp2D, lbp3D, log, square root, square, logarithm, exponential) were used to further analyze the features of CT and PET images in high dimension.

### Deep Learning Feature Extraction

**FIGURE 1:**
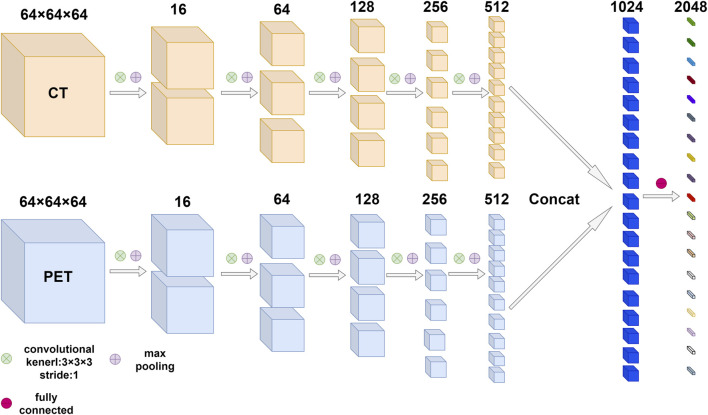
The architecture of 3D convolutional neural network based on PET/CT images. The network was composed of multiple convolution layers and max pooling layers, the number of output channels after each convolution and max pooling was marked above feature maps. Finally, the information from two modes entered the fully connected layer.

According to the characteristics of our data, a two-channel 3D convolutional neural network (3D CNN) was designed to extract deep learning features. The architecture of the 3D convolutional neural network was shown in Figure 1, each channel had five convolution layers and five max-pooling layers. The information of the two channels will eventually enter the fully connected layer. CT and PET ROIs were resized with dimension size equal to 512×512×274 into three-dimensional matrix with “simpleItk” and “numpy”, two packages of Python’s third-party library. The input of 3D CNN should only include 3D CT and PET images of the tumor area. Therefore, according to the tumor ROI, a rectangular bounding box was used to mark the tumor area, which covered the primary tumor area of NSCLC. To reduce the possible impact of a few areas outside the tumor contained in the cuboid bounding box on the model, the cuboid bounding box was divided into multiple small cuboid bounding boxes of 64×64×64 . All small cuboid bounding boxes containing more than 90% of the tumor volume were input into 3D CNN to extract the deep learning features of each patient. The deep learning framework was based on paddlepaddle (https://www.paddlepaddle.org.cn). The image matrices of the two modes were input into the corresponding channels of the 3D CNN. Finally, 2048 features for each patient output from the fully connected layer were used as deep learning features.

### Feature Selection and Model Establishment

The number of radiomics features in our study was larger than that of patients, so it may lead to the overfitting of the radiomics model. To avoid the reduction of the model classification ability, three steps were used to reduce dimenality. Firstly, Mann-Whiteney U test was used to exclude the features with the *p*-value > 0.05. Then the least absolute shrinkage and selection operator (LASSO) algorithm was used to find out the most significant features with nonzero coefficients based on 10-fold cross-validation. Finally, by combining the features selected from the LASSO regression and corresponding coefficients, the bimodal radiomics score (RS) was calculated for every patient in the training set and validation set. Then multivariate logistic regression was used to establish the radiomics model (RM). It is obvious that dimensionality reduction was also required for building the deep learning model (DLM). By repeating the above three steps, every patient’s deep learning score (DS) was calculated and the DLM model was established. In addition, multivariable logistic regression was conducted on the selected clinical characteristics with statistical significance (*p*-value < 0.05) combined with DS, and established a hybrid model (HM).

### Statistical Analysis

**FIGURE 2:**
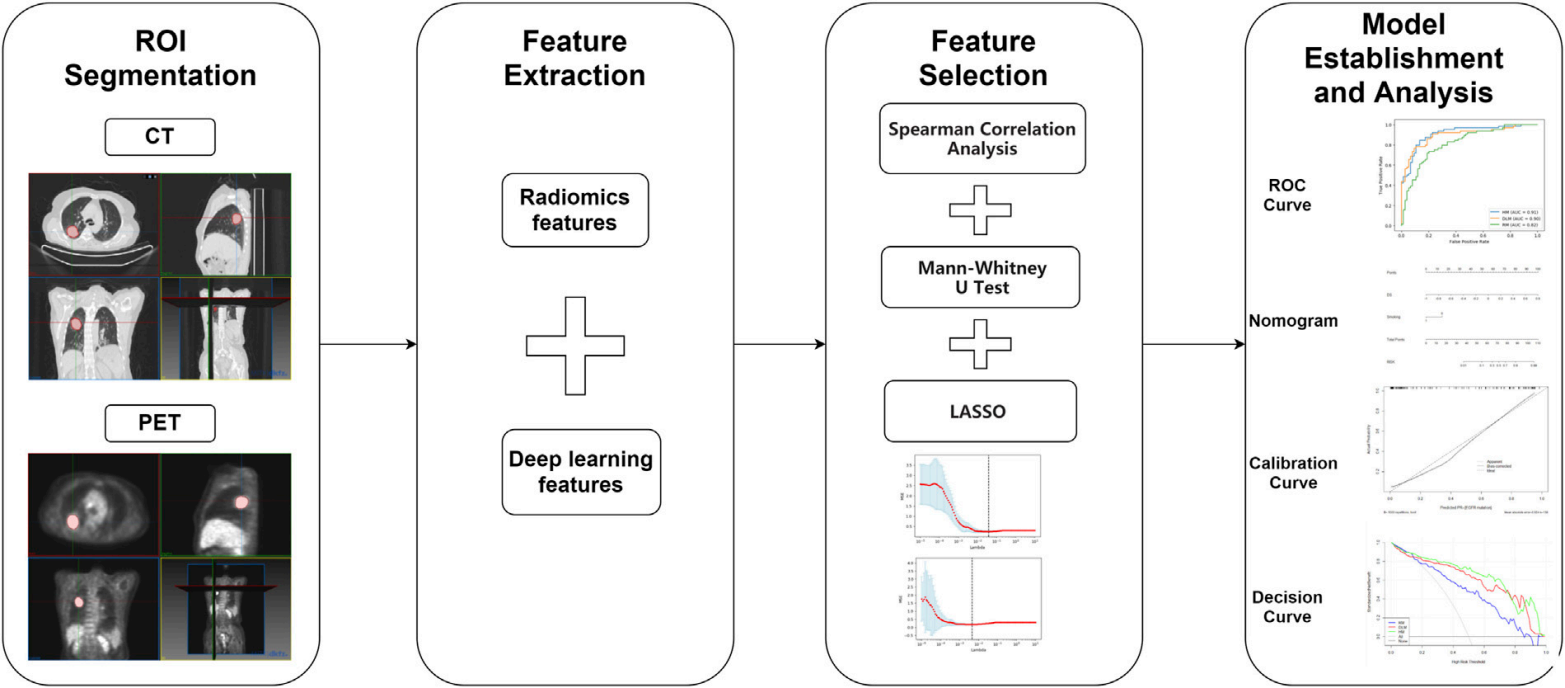
The workflow of this study.

For the clinical characteristics of patients with different EGFR mutation statuses in the training set and validation set, different test methods were used to evaluate, to find the characteristics with significant differences. For continuous variables (age, CEA, SUVmax), Mann-Whiteney U-test was used, and for categorical variables, the chi-square test was used. Clinical characteristics with two-sided *p* values <0.05 were considered to have significant differences. The third-party open-source library “pROC” of R language (https://www.r-project.org/) was used to draw ROC curves, the “rms” package was used to draw calibration curves and a nomogram, and the “rmda” package was used to draw decision curves. In addition, the sensitivity, specificity, and accuracy of different models were calculated in the training set and validation set to evaluate their performance. The ROC curves were compared and analyzed by the Delong test. The workflow of this study can be represented by the following Figure 2.

## Results

### Clinical Characteristics

The statistics of clinical characteristics of patients were shown in Table 1. In the training set, there was no significant difference in CEA, Age and SUVmax between patients in the EGFR mutation group and patients in the non-EGFR mutation group. The *p* values of smoking and gender were <0.05. In the validation set, only the *p* value of smoking was <0.05.

**TABLE 1:** Clinical characteristics for patients in the training set and validation set.

Characteristic	Training set (*n* = 138)		*p*	Validation set (*n* = 57)		*p*
	EGFR− (*n* = 74)	EGFR+ (*n* = 64)		EGFR− (*n* = 25)	EGFR+ (*n* = 32)	
Age (mean ± SD)	62.54 ± 11.26	58.50 ± 10.48	0.057	59.33 ± 10.76	64.18 ± 11.15	0.076
Gender			<0.001			0.084
Male	60 (80.18%)	28 (43.75%)		19 (76%)	16 (50%)	
Female	14 (18.92%)	36 (56.25%)		6 (24%)	16 (50%)	
Smoking			<0.001			0.043
Yes	57 (77.03%)	42 (65.62%)		8 (32%)	20 (62.50%)	
No	17 (22.97%)	22 (34.38%)		17 (68%)	12 (37.50%)	
CEA (ng/ml)	4.57 (2.99,6.52)	5.97 (3.20,20.44)	0.343	4.60 (3.14,10.35)	5.13 (2,65,18.28)	0.871
SUVmax	8.31 (6.12,12.36)	10.24 (4.96,14.65)	0.641	9.97 (6.47,14.61)	9.36 (5.97,13.54)	0.782

SD, standard deviation; CEA, carcinoembryonic antigen.

### Feature Selection and Model Establishment

4306 radiomics features were respectively extracted from both PET and CT images using pyradiomics, as described in the Supplementary Table S1. All features comply with IBSI standards. After feature selection, 419 radiomics fearures were left after Mann-Whiteney U test and 11 features were finally retained after lasso regression. Among them, six features were from PET images, and five features were from CT images. According to the coefficients corresponding to these 11 features after lasso regression, the radiomics score (RS) of every patient was obtained. Then, radiomics model (RM) was established by logistic regression based on RS.

$$\begin{array}{l} \text{RS}=\left(-0.004932\right)\times \text{PET}\_\text{gradient}\_\text{glszm}\_\text{ZoneEntropy}+0.008102\\ \times \text{PET}\_\text{wavelet}\_\text{HLH}\_\text{glrlm}\_\text{GrayLevelNonUniformityNormalized}\\ +\left(-0.100741\right)\times \text{PET}\_\text{lbp}\_2\text{D}\_\text{glcm}\_\text{ClusterProminence}+0.066754\\ \times \text{PET}\_\text{wavelet}\_\text{LHH}\_\text{glszm}\_\text{SizeZoneNonUniformityNormalized}+\left(-0.024189\right)\\ \times \text{CT}\_\text{log}\_\text{sigma}\_1\_0\_\text{mm}-3\text{D}\_\text{glszm}\_\text{SmallAreaEmphasis}+\left(-0.036134\right)\\ \times \text{CT}\_\text{gradient}\_\text{glszm}\_\text{SizeZoneNonUniformityNormalized}+0.028689\\ \times \text{CT}\_\text{wavelet}-\text{HHL}\_\text{glszm}\_\text{LargeAreaHighGrayLevelEmphasis}+0.016727\\ \times \text{PET}\_\text{wavelet}\_\text{LLH}\_\text{glszm}\_\text{SmallAreaLowGrayLevelEmphasis}+\left(-0.021518\right)\\ \times \text{CT}\_\text{square}\_\text{glrlm}\_\text{RunEntropy}+\left(-0.038129\right)\\ \times \text{CT}\_\text{original}\_\text{firstorder}\_\text{Maximum}+\left(-0.026901\right)\times \text{PET}\_\text{wavelet}\_\text{HLH}\_\text{glszm}\_\text{ZoneEntropy}+0.5 \end{array}$$

**FIGURE 3:**
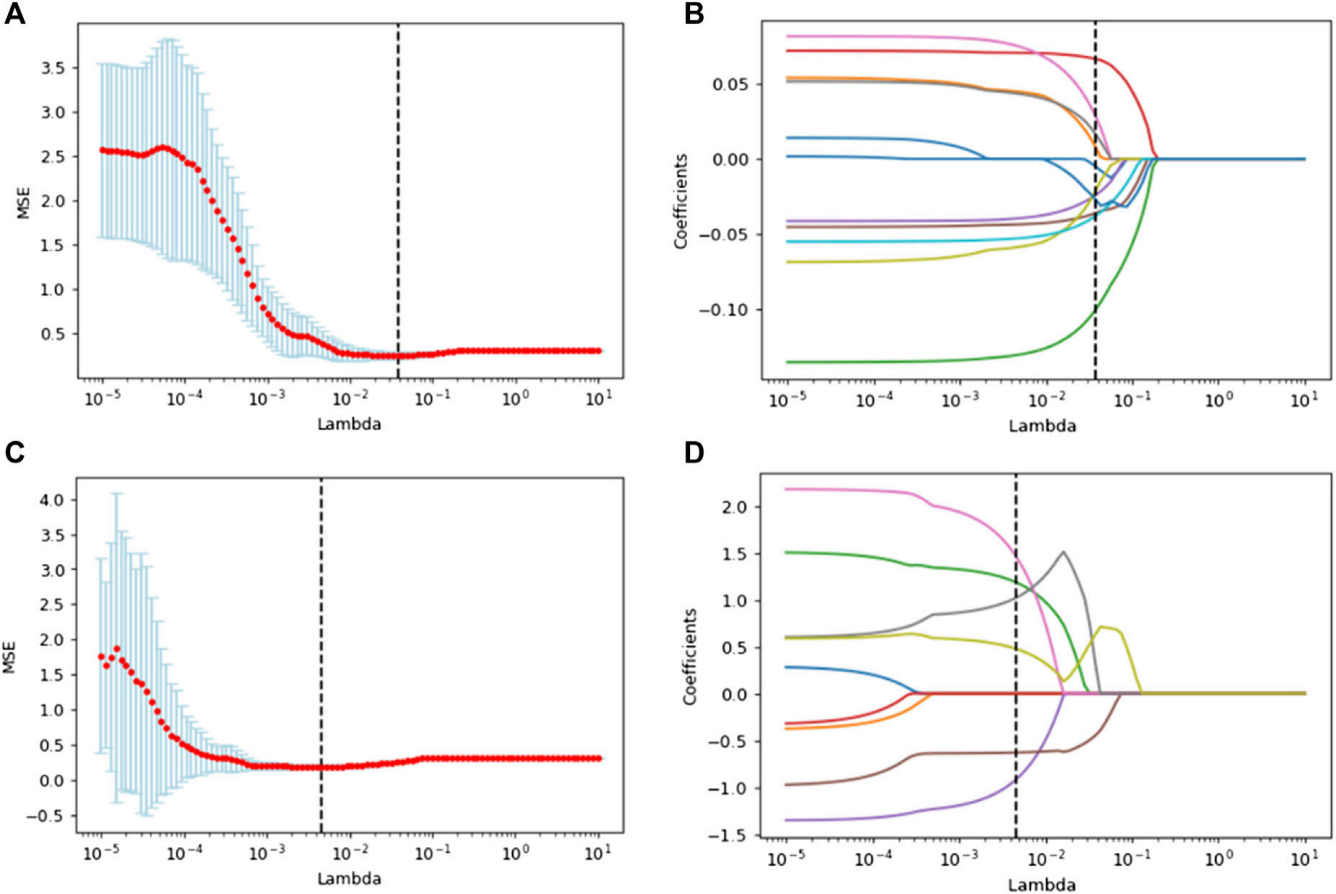
Lasso regression was used to select the optical radiomics features **(A)** and deep learning features **(C)**. **(B)** and **(D)** showed lasso coefficients of selected radiomics features and deep learning features respectively.

Since the 2048 deep learning features output by the fully connected layer of 3D CNN were not artificially defined and had specific names, these features were numbered. After feature selection, a total of 579 features were left after, the process of LASSO regression of radiomics features and deep learning features and their final retained features were shown in Figure 3. After Mann-Whiteney U test and nine features constituted the deep learning score (DS) of each patient, and the remaining 2039 features were excluded and logistic regression was used to establish a deep learning model (DLM) based on DS. After Mann-Whitney U test, in the training set, the *p* value of RS and DS for patients with different EGFR status was less than 0.001. In the validation set, the *p* value of RS was 0.024 and the *p* value of DS was less than 0.001. The above results showed that there were significant statistical differences in RS and DS in patients with different EGFR status.

$$\begin{array}{l} \text{DS}=0.5300\times \text{DF}\_1620+0.021436\times \text{DF}\_1591+0.539910\times \text{DF}\_1862+0.019825\times \text{DF}\_657\\ +0.021543\times \text{DF}\_1931+\left(-0.034021\right)\times \text{DF}\_223+0.665785\times \text{DF}\_1469\\ +0.050838\times \text{DF}\_1370+0.538539\times \text{DF}\_462+0.3753 \end{array}$$

### Performance Comparison of Three Models

**FIGURE 4:**
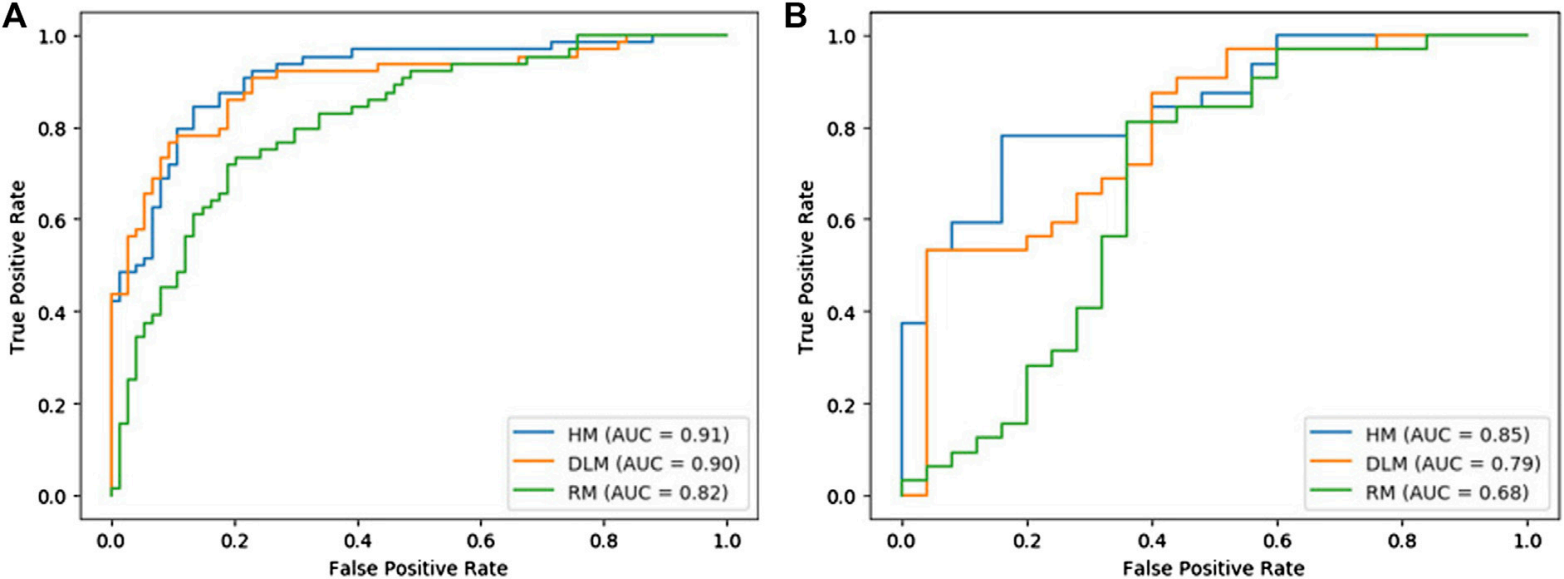
Roc curves of three models in the training set **(A)** and validation set **(B)**.

In Table 2, the performance of each model was listed. Figure 4 showed the comparison of ROC curves of three models. In the training set and validation set, the AUC values of RM were 0.82 and 0.68 respectively, and the accuracy was 0.77 and 0.74 respectively. The AUC value of DLM in training set (0.90, 95% CI: 0.85–0.95) and validation set (0.79, 95% CI: 0.67–0.91) was higher than that of RM in training set (0.82, 95% CI: 0.75–0.89) and validation set (0.68, 95% CI: 0.52–0.84). But the accuracy of DLM in the validation set (0.72) was lower than that of RM (0.74). The sensitivity and specificity of the two models were all high in the training set and validation set.

**TABLE 2:** Auc, specificity, sensitivity, and accuracy for three models in the training set and validation set.

	RM training	RM validation	DLM training	DLM validation	HM training	HM validation
Auc (95% CI)	0.82 (0.75–0.89)	0.68 (0.52–0.84)	0.90 (0.85–0.95)	0.79 (0.67–0.91)	0.91 (0.86–0.96)	0.85 (0.77–0.93)
Specificity	0.80	0.64	0.77	0.96	0.85	0.88
Sensitivity	0.73	0.81	0.91	0.53	0.89	0.78
Accuracy	0.77	0.74	0.83	0.72	0.87	0.82

Because the AUC value of DLM was higher than that of RM, to explore whether a model with better performance could be obtained, smoking was combined with DS for multivariable logistic regression to establish a hybrid model (HM). The AUCs of HM were higher than those of RM and DLM both in the training set and validation set, which were 0.91 (95% CI: 0.86–0.96) and 0.85 (95% CI: 0.77–0.93) respectively. In the validation set, the accuracy of HM was 10% higher than that of DLM (0.72), which was 0.82.

Delong test was conducted among three models. The *p* values between RM and DLM were 0.022 and 0.089, the *p* values between the RM model and HM model were 0.003 and 0.008, and the *p* values between the DLM model and HM model were 0.258 and 0.117. Although the results of the Delong test showed that there was no significant difference between the ROC curves of HM and DLM, HM showed excellent diagnostic performance.

**FIGURE 5:**
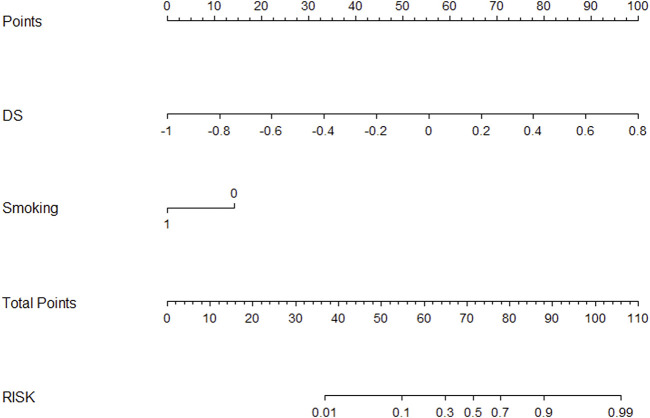
A nomogram based on HM.

**FIGURE 6:**
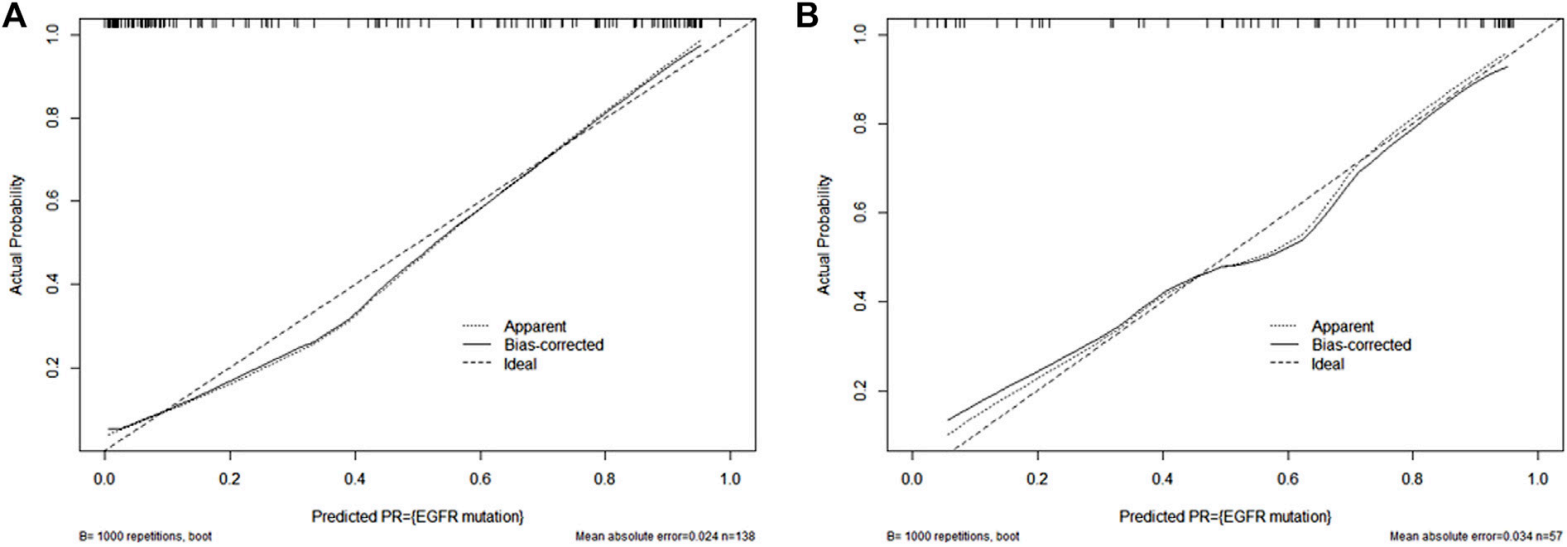
HM’s calibration curves in the training set **(A)** and validation set **(B)**.

**FIGURE 7:**
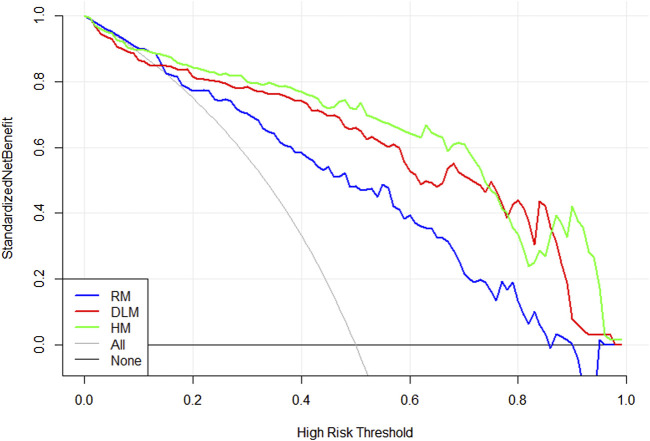
A decision curve was plotted to show the standardized net benefit of three models.

Figure 5 showed a nomogram based on HM. Doctors can judge the possibility of EGFR mutation status more intuitively and conveniently according to the relevant information of patients according to the nomogram. Figure 6 showed the calibration curves of HM. It could be seen that the deviation between the model-predicted probability and the expected probability was small as a whole. Figure 7 showed the decision curves analysis (DCA) of three models. It could be seen that the clinical benefit obtained from RM was the lowest, while the clinical benefit obtained from HM was the highest.

## Discussion

In this study, PET/CT images of 194 patients were used with radiomics and a deep learning model based on 3D CNN to predict the EGFR mutation status of NSCLC patients. The AUC values of the three models established ranged from 0.82–0.91 in the training set and from 0.68 to 0.85 in the validation set. The results showed that the AUC value of DLM was higher than that of RM in both the training set and verification set. The accuracy of RM was only 0.02 higher than that of DLM in the validation set but 0.05 lower than DLM in the training set. In addition, the clinical characteristics of patients in the training set and validation set were analyzed and selected smoking as a statistically significant factor. Then based on the deep learning score (DS) and smoking of each patient, the hybrid model (HM) was produced. This model achieved the largest AUC value (0.91 95% CI: 0.86–0.96 for the training set and 0.85 95% CI: 0.77–0.93 for the validation set) and the highest accuracy rate (0.87 for the training set and 0.82 for the validation set). Although the results of the Delong test showed that there was no difference between the ROC curves of DLM and HM, HM achieved excellent results in predicting EGFR mutation status in patients with NSCLC. In the training set and validation set, the AUC value and accuracy of HM were all higher than RM and DLM. The drawn decision curve also showed that HM obtained the greatest clinical benefit. Finally, a nomogram based on HM was also drawn to help doctors more conveniently judge the possibility of EGFR mutation in patients with NSCLC.

Radiomics can extract a large number of image features quickly from various modal medical images, and researchers can use a variety of filters to obtain the features of these medical images in higher dimensions. Thereafter, a variety of dimensionality reduction methods can be used to find the feature subset most related to gene mutation. Previous studies have shown that radiomics can accurately judge the EGFR mutation status of NSCLC patients. The deep learning model represented by a convolutional neural network has a variety of hierarchical structures, which can carry out various linear and nonlinear transformations on the original image, to find more potential features in the image. Compared with radiomics, deep learning used to obtain higher accuracy in predicting EGFR mutations in patients, which has also been proved by our research (Kirienko et al., 2018; Hassani et al., 2019; Kang et al., 2019; Sollini et al., 2019; Tu et al., 2019; Jiang et al., 2020). Previous studies mostly used radiomics or deep learning based on unimodal images (CT/MRI) (Li et al., 2018; Digumarthy et al., 2019; Lu et al., 2020; Wu et al., 2020; Zhang B. et al., 2020), and other studies have shown that using multimodal medical images can mine more information related to patients’ gene mutations to improve the accuracy of prediction and provide more guidance for patients’ future treatment plans (Mu et al., 2020; Zhang J. et al., 2020; Zhang M. et al., 2020; Chang et al., 2021). To compare radiomics and deep learning in predicting EGFR mutation status in patients with NSCLC based on PET/CT images, the two-channel deep learning model and radiomics model based on PET/CT images were designed, and the prediction performance of them were compared. In the selection of clinical factors, as in previous studies, smoking has been proved to be a variable closely related to EGFR mutation status in patients with NSCLC (Ren et al., 2021; Yin et al., 2021). The HM model based on DS and smoking showed excellent performance and potential of combining significant clinical factors and deep learning system based on multimodal images in such research.

This study also had some limitations. First, the collected patient data were from a single-center and are all Chinese. In the future, research in more centers should be conducted and data from different races should be collected for further analysis. Second, due to the relatively limited size of our data set, the accuracy of DLM model and HM model based on our neural network may be high. Larger sample size should be needed in future studies. Third, as a black box, the logic and process of deep learning still need more research to improve interpretability, and more methods should be explored to solve this problem in the future. Fourth, there were only five clinical characteristics of patients collected in this study, more clinical information about patients with NSCLC will be obtained to study whether a better model can be established.

## Data Availability

The original contributions presented in the study are included in the article/Supplementary Materials, further inquiries can be directed to the corresponding authors.
